# The Modulatory Effect of *Cyclocarya paliurus* Flavonoids on Intestinal Microbiota and Hypothalamus Clock Genes in a Circadian Rhythm Disorder Mouse Model

**DOI:** 10.3390/nu14112308

**Published:** 2022-05-31

**Authors:** Ying Sun, Chi-Tang Ho, Yanan Liu, Shennan Zhan, Zufang Wu, Xiaojie Zheng, Xin Zhang

**Affiliations:** 1 Department of Food Science and Engineering, Ningbo University, Ningbo 315211, China; sysy42365@163.com (Y.S.); liuyanan@nbu.edu.cn (Y.L.); zhanshengnan@nbu.edu.cn (S.Z.); zxdqqzyc@163.com (Z.W.); 2 Department of Food Science, Rutgers University, New Brunswick, NJ 08901, USA; 3 Department of Agriculture and Biotechnology, Wenzhou Vocational College of Science and Technology, Wenzhou 325006, China

**Keywords:** circadian rhythm disruption, *Cyclocarya paliurus* flavonoids, gut microbiota, metabolites, single-cell RNA-seq

## Abstract

Circadian rhythm disruption is detrimental and results in adverse health consequences. We used a multi-omics profiling approach to investigate the effects of *Cyclocarya paliurus* flavonoid (CPF)-enriched diets on gut microbiota, metabolites, and hypothalamus clock genes in mice with induced circadian rhythm disruption. It was observed that CPF supplementation altered the specific composition and function of gut microbiota and metabolites induced by circadian rhythm disruption. Analysis showed that the abundance of *Akkermansia* increased, while the abundance of *Clostridiales* and *Ruminiclostridium* displayed a significant downward trend after the CPF intervention. Correlation analysis also revealed that these gut microbes had certain correlations with the metabolites, suggesting that CPFs help the intestinal microbiota to repair the intestinal environment and modulate the release of some beneficial metabolites. Notably, single-cell RNA-seq revealed that CPF supplementation significantly regulated the expression of genes associated with circadian rhythm, myelination, and neurodegenerative diseases. Altogether, these findings highlight that CPFs may represent a promising dietary therapeutic strategy for treating circadian rhythm disruption.

## 1. Introduction

Every organism is adapted to an intrinsic circadian rhythm of 24 h that allows organisms to synchronize their behavior with regular and predictable routines to adjust to daily environmental changes; this rhythm also drives physiological and cellular adaptations [1,2]. The rhythms of daily life activities and a wide variety of processes, including gastrointestinal function and metabolic processes, are regulated by endogenous cellular autonomic circadian rhythm oscillation and daily eating [3]. The circadian rhythm is orchestrated by the brain’s central clock in the suprachiasmatic nucleus (SCN) of the hypothalamus, which in turn harmonizes peripheral clock genes present in peripheral organs (e.g., the liver, heart, lungs, and kidneys) and immune cells [4,5]. Clock genes (*Bmal1*, *Clock*, *Cry1*, *Cry2*, *Per1*, *Per2*, *Per3*, etc.) exhibit specific expression to produce proteins and then regulate circadian processes via self-sustaining transcriptional/translational feedback loops [6]. Nonetheless, circadian rhythm disruption is becoming increasingly apparent in contemporary society due to the widespread exposure to jet lag, shiftwork, late-night eating, and light-noise [7]. This misalignment of the rhythm is detrimental: it results in adverse health consequences and increases the risks of diseases such as metabolic syndrome, cancer, cardiovascular disease, and neurodegenerative disease [8]. Intriguingly, a large number of studies associating dietary regimens, gut microbiota changes, and circadian rhythm propel a plethora of dietary interventions aimed at promoting a “healthy microbiota” and ameliorating diseases caused by circadian rhythm disruption [9,10,11].

A growing body of in vitro and in vivo studies reveal that the gut microbiota, a newly discovered player in its host’s wellbeing and health, represents a burgeoning, fascinating, and promising area of research for understanding the development of certain diseases, as well as their treatment and prevention [12]. As a complex admixture of *Bacteroidetes*, *Firmicutes*, *Actinobacteria*, *Proteobacteria*, and *Verrucomicrobia*, the gut microbiota differentially benefits from and affects the host’s physiology [13]. Interestingly, certain members of the gut microbiota also exhibit diurnal variations in relative abundance and function and play a pivotal role in the host’s circadian network as non-canonical drivers [14]. These microorganisms have been recognized as “microbial oscillators” [15]. They entrain upon non-photic cues, primarily dietary, rather than photic cues to modulate host circadian networks and metabolism directly or indirectly via presumed small-molecule mediators [14]. Our previous research has demonstrated that oolong tea polyphenol supplementation significantly modulated the circadian rhythm oscillations of certain microbiota in mice exposed to constant dark treatment, including *Clostridium*, *Prevotella*, and *Bacteroides*, and then effectively ameliorated the circadian rhythm disorder [16].

*Cyclocarya paliurus* (Batal.) Iljinskaja (*C. paliurus*), a plant exclusively grown in the southern provinces of China, has been widely used as a traditional tea and medicine [17]. The extracts of *C. paliurus* contain triterpenoids, flavonoids, phenolic compounds, and polysaccharides and can reduce blood pressure and lipid levels, alleviate type 2 diabetic symptoms, and protect against diabetic cardiomyopathy [18,19,20]. *C. paliurus* flavonoids (CPFs) are the primary bioactive components in the leaves of *C. paliurus.* Considerable interest has arisen regarding the effects of CPFs on lowering serum lipids, blood sugar, and blood pressure, as well as improving immunity [21]. More importantly, as a result of low bioavailability, the majority of CPFs enter via the colon, where CPFs can interact with gut microbiota to promote or inhibit their growth and release beneficial metabolites [22]. Meanwhile, these manifold interactions also are advantageous to circadian rhythm disruption. We found that CPFs had prebiotic-like activity in the human-microbiota-associated mice gut; this is relevant given the importance of shaping the structure of gut microbiota and affecting microbial metabolic functions [23]. Furthermore, our previous research also showed that CPFs significantly alleviated the disrupted diurnal oscillation and phase shift of the specific intestinal microbes and liver clock genes whose expression was induced by constant darkness [5].

Although there are many reports on CPF constituents, few studies link the efficacy of CPFs with the gut microbiota and metabolite perturbation and brain dysfunction caused by circadian rhythm disruption. In this study, we used a multi-omics profiling approach to investigate the beneficial effects of CPF-enriched diets on the gut microbiota, metabolites, and brain function in mice with respect to changes induced by circadian rhythm disruption. This approach provides more information on the interplay between the gut and brain.

## 2. Materials and Methods

### 2.1. Chemicals

The leaves of *C. paliurus* were obtained from Quanshan Chinese Herbal Medicine Planting Co., Ltd. (Wenzhou, China). Polyamide resin was purchased from Ocean Chemical Co., Ltd. (Qingdao, China). All other chemicals were of analytical grade.

### 2.2. Preparation of CPFs

The CPF preparation method was based on previous research with appropriate modifications [5,23]. Detailed preparation methods are described in the Supporting Information.

### 2.3. CPF Dose Determination in Animal Experiments

We first calculated the human clinical dose equivalent to the clinical dosage of CPF based on the extraction rate of CPF (78%). The optimal dose of CPF in the present study was determined to be 200 mg/kg, and the intake of CPF was 156 mg/kg.

### 2.4. Animals and Experimental Design

Six-week-old male C57BL/6J mice from Hunan Slack Experimental Animal Co., Ltd. (Hunan, China), weighing 18–20 g, were housed individually in animal cages in a sterilized room with a controlled temperature of 22 ± 1 °C and a humidity of 55 ± 5%. The mice were allowed free access to standard rodent chow and sterile distilled water throughout the study. After a week of acclimatization under a regular light–dark cycle period, they were randomly assigned to 3 groups (15 animals in each group), including the 12 h light–dark cycle control group (CT group), the constant darkness group (CD group), and the constant darkness fed with CPF group (CPF group). Mice in the CPF group were administered 200 mg/kg CPF by gavage once a day. Mice in the CT and CD groups received 0.2 mL of sterile saline by gavage. Body weight and food consumption were recorded weekly. At the indicated intervals (1, 2, 3, and 4 weeks) during the administration period, fecal samples were collected using sterilized Eppendorf tubes and immediately stored at −80 °C for analysis of the fecal microbiota. After 4 weeks of continuous administration, all mice in the CT, CD, and CPF groups were sacrificed under pentobarbital anesthesia, and fecal samples, as well as the contents of the cecum and the hypothalamus, were collected and immediately frozen. All procedures involving animals were conducted at the Centre for Laboratory Animals, Ningbo University (Permission No. SYXK [Zhejiang] 2013-0191).

### 2.5. Intestinal Microbiota Analysis

DNA from different samples was extracted using the E.Z.N.A. ^®^Stool DNA Kit (D4015, Omega Bio-tek, Inc., Norcross, GA, USA) according to the manufacturer’s instructions. Detailed determination methods are described in the Supporting Information. α-Diversity analyses were performed using the QIIME (Quantitative Insights Into Microbial Ecology) (version 1.2.8) software package [24], and high-quality reads were collected into the operational classification unit (OTU) for a series of analyses [25].

### 2.6. Fecal and Cecum Sample Metabolite Extraction

The fecal and cecum samples were thawed on ice, and metabolites were extracted from 20 µL of each sample using 120 µL of precooled 50% methanol buffer. The mixture of metabolites was vortexed for 1 min, incubated at room temperature for 10 min, and stored at −20 °C overnight. Then, the sample was centrifuged at 4000× *g* for 20 min, and the supernatant was transferred into 96-well plates. The samples were stored at −80 °C prior to the liquid chromatography–mass spectrometry (LC–MS) analysis. Additionally, a quality control (QC) sample was also prepared by combining 10 μL of each extraction mixture to monitor the data quality.

### 2.7. Untargeted Metabolomics Analyses

LC–MS analysis of metabolic subclasses was performed on a TripleTOF 5600 Plus high-resolution tandem mass spectrometer (SCIEX, Warrington, UK) coupled with an ultra-performance liquid chromatography (UPLC) system (SCIEX, Warrington, UK). Detailed determination methods are described in the Supporting Information. Furthermore, a QC sample was analyzed every 10 samples to evaluate the stability of the LC–MS. Finally, the acquired LC–MS data pretreatment was performed using XCMS software.

### 2.8. Single Cell Preparation and scRNA-seq

Single cells were captured in the Chromium Controller (10X Genomics), and RNA-seq libraries were prepared following the manufacturer’s protocol [26]. Detailed determination methods are described in the Supporting Information. The libraries were subjected to high-throughput sequencing using the Chromium Single Cell 30 Reagent Kit (v2 Chemistry).

### 2.9. Bioinformatic Analysis of scRNA-seq Data

To identify unbiased cell types of scRNA-seq, we annotated the cell clusters using single-cell transcriptomics R and compared them with the Immunological Genome Project (reference mouse dataset) [27]. Enrichment analysis was performed according to previous methods [28,29].

### 2.10. Statistical Analysis

Data obtained were analyzed by SPSS v 17.0.0 (SPSS Inc., Chicago, IL, USA) and expressed as mean ± standard deviation (SD) for at least three replicates. Significant differences between groups were analyzed by one-way analysis of variance (ANOVA), and the post hoc Tukey test was used for multiple comparisons. All results were considered statistically significant at *p*-values < 0.05.

## 3. Results

### 3.1. The Contents of CPF in the Extract

The present study demonstrated that the predominant flavonoid in the leaves of *C. paliurus* was kaempferol-3-O-β-glucuronide (363.78 ± 15.23 mg/g). Additionally, the contents of kaempferol-3-O-α-L-rhamnopyranoside, isoquercitrin, and quercetin in the CPF extract were 208.45 ± 9.28, 128.46 ± 7.18, and 83.66 ± 3.67 mg/g, respectively.

### 3.2. CPF Supplementation Improves the Perturbation of Gut Microbiota in Mice with Circadian Rhythm Disturbance

**Figure 1:**
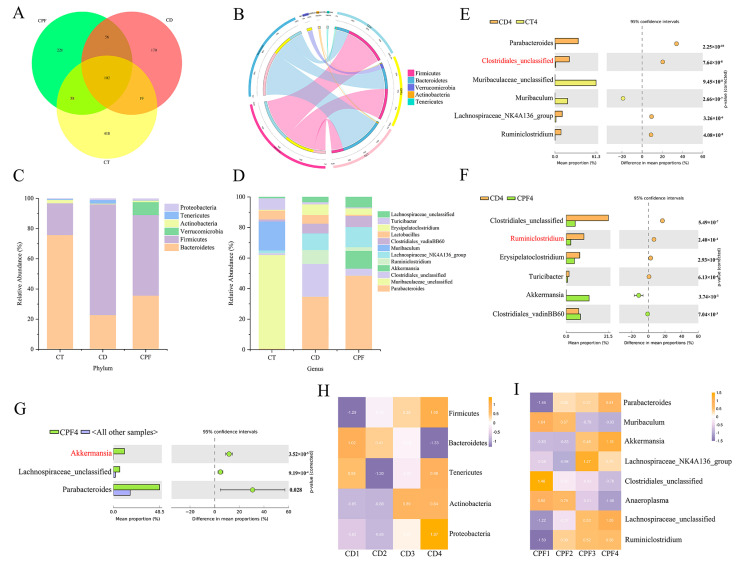
The effect of CPF intervention on intestinal microbiota in mice with circadian rhythm disturbance: (**A**) Venn diagrams of the operational taxonomic unit (OTU) in the three groups. (**B**) Circos plot of the top5 microbial taxa at the phylum level. Microbial distributions of different groups at the phylum (**C**) and genus levels (**D**). Difference analysis of gut microbiota composition at the genus level between the CD and CT groups (**E**) and between the CD and CPF groups (**F**). (**G**) Dominant microbial taxa of the CPF group. (**H**) The alternation of the microbial taxa in the CD group. (**I**) The alternation of the microbial taxa in the CPF group.

In our previous study, we demonstrated that a constant darkness intervention could significantly contribute to the perturbation of the gut microbiota in a mouse model [5]. Here, we further analyzed relative abundances at the phylum and genus levels to confirm the effect of CPF intervention on intestinal microbiota in mice with circadian rhythm disturbance. The Venn diagram shows the shared and unique OTUs among the three groups (Figure 1A), of which 102 shared OTUs were detected in all samples. The OTUs and Chao1 in the CD group were significantly lower than in other groups (*p* < 0.05), and both the Shannon and Simpson indexes showed that the fecal microbial diversity of the CPF group was significantly higher than that of the CD group (*p* < 0.05) (Table 1).

At the phylum level, the major microbial communities were *Bacteroidetes*, *Firmicutes*, *Verrucomicrobia*, *Actinobacteria*, and *Tenericutes* (Figure 1B). As shown in Figure 1C, the relative abundance of *Bacteroidetes* showed a significant downward trend after circadian rhythm disturbance for 4 weeks compared with the CT group, while the relative abundance of *Firmicutes* was of the opposite trend (*p* < 0.001) (Figure S1A). Interestingly, CPF treatment increased the abundance of *Bacteroidetes* and decreased the abundance of *Firmicutes* (*p* < 0.001) (Figure S1B). These major differences in the two most abundant OTUs led to a significant decrease in the *Firmicutes*/*Bacteroidetes* (F/B) ratio in the CPF and CD groups, being 1.50 and 3.23, respectively (*p* < 0.001). Additionally, our results also demonstrated that CPF intervention facilitated the growth of *Verrucomicrobia*, indicating that CPF intervention effectively inhibited the growth of *Firmicutes* and benefitted the stability of certain intestinal microbiota, especially in this environmentally triggered microbial imbalance.

At the genus level (Figure 1D), after CPF supplementation, the microbial composition changed. Results revealed that circadian rhythm disturbance reduced the abundance of *Muribaculaceae* and increased the abundance of *Parabacteroides*, *Clostridiales*, *Ruminiclostridium*, and *Lachnospiraceae* significantly (*p* < 0.001) (Figure 1E). Conversely, the relative abundance of *Clostridiales* and *Ruminiclostridium* were significantly reduced under CPF intervention for 4 consecutive weeks (*p* < 0.001) (Figure 1F), which was highly consistent with the CT group. Notably, the relative abundance of *Akkermansia* displayed a significant upward trend (*p* < 0.001), displaying distinctive characteristics different from the other groups (Figure 1G).

Based on the above findings, we deeply analyzed the alternation of the abundance of *Bacteroidetes* and *Firmicutes* during circadian rhythm disruption. Results showed the abundance of *Firmicutes* increasing with 4 weeks of continuous circadian rhythm disruption, while the abundance of *Bacteroidetes* exhibited the opposite trend (Figure 1H). Meanwhile, we also noticed that the relative abundance of *Akkermansia* increased only in the CPF group (Figure 1I and Figure S1C). These results indicated that CPFs might have the potential to shape the abundance of significant microbiota and improve the perturbation of intestinal microbiota caused by circadian rhythm disorders.

### 3.3. Characterization of Metabolic Differences

The analysis of non-targeted metabolomics showed a significant difference between the CD group and the CPF group. The unsupervised principal component analysis (PCA) is mainly used to observe the trend of separation between groups in experimental models and to reflect the variability between groups from the original data (Figure S2A). Meanwhile, there were significant differences between the CD and CPF groups, with PC1 at 25.21% and PC2 at 13.03%, which indicated that CPF intervention influenced the metabolism in mice with circadian rhythm disruption (Figure S2B).

In contrast to the PCA, the supervised PLS-DA analysis is mainly applied in screening metabolic markers and the accuracy of differential metabolites. An R2 value close to 1 indicated that the quality of the model was good, and Q2 < 0 implied that the analysis of differential metabolites was accurate. As shown in Figure S2C, the metabolites of the CD group and the CPF group were also significantly different in the supervised mode, indicating that the circadian rhythm disorder model was successfully modeled. Q2 < 0 indicated that the model did not display over-fitting, and the analysis of differential metabolites was more accurate.

### 3.4. Effects of CPFs on Gut Metabolites in Mice with Circadian Rhythm Disturbance

**Figure 2:**
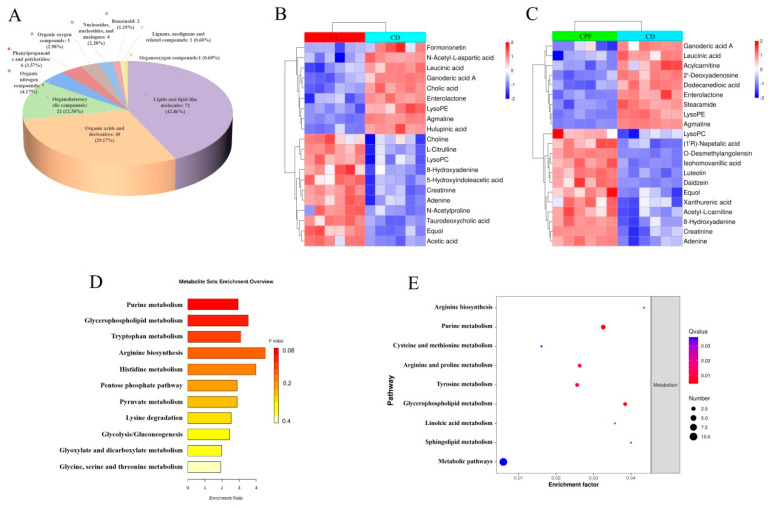
The effect of CPFs on gut metabolites in mice with circadian rhythm disturbance. (**A**) The classification of differential metabolites in the CD group. (**B**) Differential metabolites between the CT and CD groups. (**C**) Differential metabolites between the CPF and CD groups. (**D**) KEGG enrichment pathways of significantly regulated differential metabolites between the CD and CT groups. (**E**) KEGG enrichment pathways of significantly regulated differential metabolites under CPF supplementation.

Metabolome analysis revealed a substantial difference in the contents of thousands of gut metabolites. The volcano distribution diagrams of different metabolites in the CD group and the CT group are shown in Figure S2D. Compared with mice exposed to 12 h of normal light, there were 65 metabolites significantly down-regulated and 103 metabolites significantly up-regulated in CD group. In addition, the classification of differential metabolites revealed that 42.86% of the compounds were lipids and lipid-like molecules, 29.17% were organic acids and their derivatives, and 12.50% were organoheterocyclic compounds (Figure 2A). Among all metabolites determined, those that play important roles in the nervous system and metabolic diseases and that varied between groups were selected based on the variable importance in the projection (VIP). Results revealed that 9 metabolites were up-regulated and 11 metabolites were down-regulated; they are listed and clustered in Figure 2B. Different metabolites that were up-regulated included formononetin, N-acetyl-L-aspartic acid, ganoderic acid A, cholic acid, enterolactone, acylcarnitine, lysophosphatidylethanolamine (lysoPE), agmatine, and others. Different metabolites that were down-regulated included choline, L-citrulline, lysophosphatidylcholine (lysoPC), 8-hydroxyadenine, 5-hydroxyindoleacetic acid, creatinine, adenine, N-acetylproline, taurodeoxycholic acid, equol, acetic acid, and others. Our results showed that important metabolites derived from the interaction between host and gut microbiota, such as equol, agmatine, N-acetyl-L-aspartic acid, lysoPE, lysoPC, choline, and 8-hydroxyadenine exhibit the characteristics of disorder in mice with circadian rhythm disruption. Actually, these metabolites have a unique role in maintaining homeostasis in the brain, heart, and surrounding vascular structure. In some instances, agmatine has cellular protective effects and contributes to cell proliferation and differentiation in the CNS, which can act as potential neuroprotection for the treatment of acute brain disorders and spinal cord injury [30].

Having shown that circadian rhythm disruption influences the gut metabolites of healthy mice, we next sought to analyze the dynamic changes in potential differential metabolites between the CD group and the CPF group. The volcano distribution diagrams of different metabolites in the CD and CPF groups are shown in Figure S2E. Consistently, we sorted the metabolites in the CPF group compared with the CD group based on the descending order of the VIP value. Our results demonstrated that a total of 21 differential metabolites were significantly regulated, including 12 up-regulated and 9 down-regulated metabolites (Figure 2C). CPF supplementation increased the levels of several metabolites, including lysoPC, equol, acetyl-L-carnitine, 8-hydroxyadenine, creatinine, adenine, and others. In addition, the down-regulated metabolites in the CPF group were ganoderic acid A, acylcarnitine, enterolactone, lysoPE, agmatine, and others. It is worth noting that CPFs can reverse the changes in relative metabolite levels caused by circadian rhythm disturbance under CD conditions, such as lysoPE, lysoPC and equol, which are excellent indicators of the nutritional phenotype associated with an increased risk of cardiovascular and neurodegenerative diseases.

To gain a comprehensive understanding of the important enrichment pathways involved with different metabolites, the pathway analysis of the Kyoto Encyclopedia of Genes and Genomes (KEGG) database was exploited. Our results showed that the pathways that regulated the differential metabolites affected after the constant darkness intervention mainly included the “purine metabolism”, “glycerophospholipid metabolism”, and “tryptophan metabolism” (Figure 2D). Interestingly, as is shown in Figure 2E, CPF supplementation in mice living in constantly dark conditions markedly increased the level of partial metabolites and then regulated differential metabolite enrichment pathways, which included the “purine metabolism”, “arginine biosynthesis”, and “glycerophospholipid metabolism”.

### 3.5. Correlations between the Fecal Microbial Taxa and Intestinal Differential Metabolites

**Figure 3:**
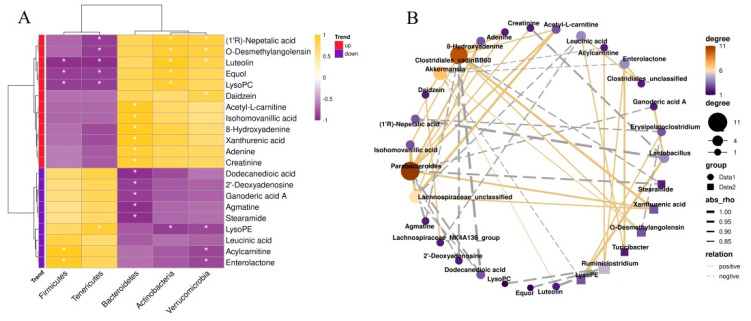
Spearman analysis of the correlation between intestinal microbial composition and intestinal differential metabolites: (**A**) Spearman correlation analysis between genus and metabolite concentrations affected by CPF supplementation. (**B**) Network diagram of intestinal differential metabolites and microbial taxa. * *p* < 0.05.

Considering that CPF intervention caused more significant changes in the intestinal microbiota of mice with circadian rhythm disturbance, a Spearman’s correlation analysis was performed to identify the correlation between the gut microbiota composition and the intestinal differential metabolites that were significantly affected by CPF supplementation. As shown in Figure 3A, we analyzed the correlation between the top5 microbial taxa enriched at the phylum level and 21 different metabolites identified in the CPF-treated mice. It is noteworthy that our results suggested that most of the up-regulated metabolites were positively correlated with the abundance of *Bacteroidetes* and *Verrucomicrobia* but negatively correlated with the abundance of *Firmicutes*. Acetyl-L-carnitine, isohomovanillic acid, 8-hydroxyadenine, xanthurenic, adenine, and creatinine were more related to *Bacteroidetes* (*p* < 0.05) (Figure 3A). In addition, daidzein, a polyphenolic compound that exhibits a preventive effect on cognitive function decline and neurodegenerative disorders, was positively correlated with *Verrucomicrobia* (*p* < 0.05).

In order to further characterize the correlation, we performed further analysis at the genus level. We mainly focused on the microbial taxa regulated by CPFs: *Akkermansia*, *Clostridiales*, and *Ruminiclostridium*. As shown in Figure 3B, the correlation analysis revealed that the genus *Akkermansia*, which increased in abundance in the CPF group, was positively correlated with daidzein, luteolin, (1’R)-nepetalic acid, and o-desmethylangolensin but negatively associated with acylcarnitine, enterolactone, and lysoPE (*p* < 0.05). In addition, the genus *Clostridiales*, which in decreased abundance in the CPF group, showed a positive correlation with enterolactone and leucinic acid (*p* < 0.05). Another important genus, *Ruminiclostridium*, presented positive associations with enterolactone and acylcarnitine but negative associations with lysoPC, equol, and luteolin (*p* < 0.05). Our data revealed that the intestinal microbiota that increased (*Akkermansia*) and decreased (*Clostridiales* and *Ruminiclostridium*) in CPF intervention had a certain correlation with the metabolites, suggesting that CPFs propelled the intestinal microbiota to repair the intestinal environment and modulate the release of some beneficial metabolites. Importantly, we noticed that CPF treatment regulated several metabolites related to myelination in brain cells and associated with neuroprotective effects, such as acetylcarnitine and daidzein. This finding provides a foundation for us to explore the effect of CPF supplementation on changes in brain cell function induced by circadian rhythm disruption.

### 3.6. Single-Cell RNA-seq Identified Circadian-Rhythm-Associated Brain Cell Populations in the Hypothalamus of Mice

**Figure 4:**
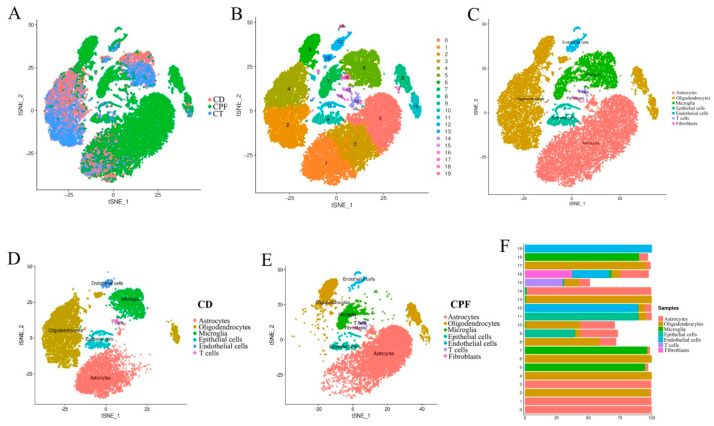
Single-cell RNA-seq identified circadian-rhythm-associated brain cell populations in the hypothalamus of mice: (**A**) t-SNE map of single cells of the hypothalamus in the CD, CT, and CPF groups. (**B**) t-SNE images of total hypothalamic cells in different clusters. (**C**) t-SNE identification map of hypothalamic single cells in the CT, CD, and CPF groups of samples. (**D**) t-SNE identification map of hypothalamic single cells in the CD group. (**E**) t-SNE identification map of hypothalamic single cells in the CPF group. (**F**) The percentage of cell types identified per cell cluster.

To compare cell types in the hypothalamus of mice, all significant cells were pooled together, and their transcriptome profiles were subjected to t-distributed stochastic neighbor embedding (t-SNE) graph-based clustering. The results of different samples and subgroups of cells are shown in Figure 4A,B. Each dot in the image represents a cell. Furthermore, the subgroups’ structural differences for each group are shown in Figure S3A–C. The results show that there were 18 clusters in the CD group and 19 clusters in the CPF group. It was found that there were significant differences between the CD and CPF groups in Cluster0, Cluster1, Cluster2, Cluster3, Cluster4, Cluster5, Cluster6, Cluster7, and Cluster8. Compared with the CD group, the cell number of the CPF group in Cluster0, Cluster3, Cluster6, Cluster7, and Cluster8 showed an up-regulated trend, whereas it showed a down-regulated trend in Cluster1, Cluster2, Cluster4, and Cluster5. These differential variations lay a foundation for us to deeply probe the clusters unique to CPFs for cell-type heterogeneity identification and the differential variation of brain cell expression caused by circadian rhythm disturbance.

Based on cell marker gene identification and interrogating the expression patterns of known gene markers, seven known cell types of the human brain were identified and annotated in three groups: astrocytes, oligodendrocytes, microglia, epithelial cells, endothelial cells, T cells, and fibroblasts (Figure 4C) [31]. Classification of the cells revealed distinct cell compositions in the CD and CPF groups. As shown in Figure 4D,E, the cell types of the two groups of samples were annotated, and the heterogeneity of the expression of each cell type in the two groups of samples could be clearly and intuitively seen. Fibroblasts only existed in the CPF group and not in the CD group. Strikingly, astrocytes were more highly enriched in the CPF group than in the CD group, at 65.24% and 35.87%, respectively. However, oligodendrocytes were more plentiful in the CD group, making up 43.38% of the total cells, while only representing 17.26% in the CPF group.

Moreover, each cell cluster contained a variable number of cells and variable transcriptional activity. We next collapsed the pre-clusters into seven broad cell type clusters using annotations supported by direct marker gene expression; thereby, we attributed clusters to their putative identities (Figure 4F). Of note, cluster0, cluster1, cluster3, and cluster14 were identified as astrocytes, which are integral to normal brain function and potentially provide a promising target for the preclinical diagnosis of nervous system diseases [32]. In addition, cluster2, cluster4, cluster6, cluster13, and cluster17 were identified as oligodendrocytes. Oligodendrocytes are the myelinating cells of the central nervous system (CNS). Additionally, viable oligodendrocytes and an intact myelin sheath are indispensable for neuronal health [33,34]. Cluster5, cluster7, and cluster18 were identified as microglia, which are essential for maintaining the health and normal function of the CNS [35]. In addition, as an immune cell in the brain, T cells accounted for the majority in cluster15. It has become evident that T cells play a critical role in resolving damage after CNS injury [36]. The identified subgroups were not exclusively enriched with cells from any single individual. In cluster16, 37.21% were identified as fibroblasts, 28.68% were endothelial cells, 22.48% were astrocytes, and 6.98% were oligodendrocytes.

### 3.7. Effects of CPFs on the Expression of Circadian Clock Genes in the Hypothalamus

**Figure 5:**
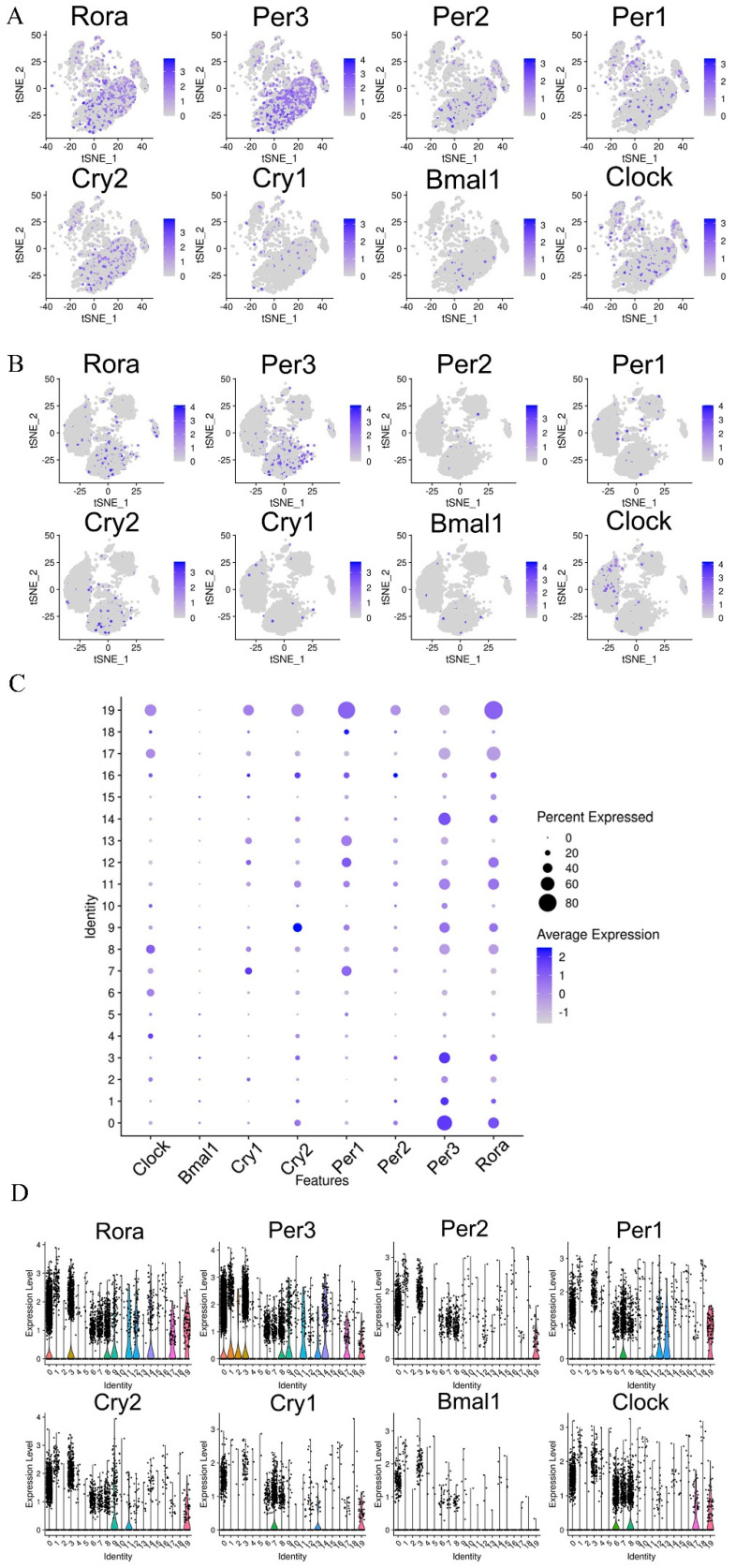
Effect of CPFs on the expression of circadian clock genes in the hypothalamus: t-SNE map of the expression of rhythm genes in the hypothalamus in the CPF group (**A**) and CD group (**B**). (**C**) Dot plots of circadian rhythm gene expression levels in different clusters of the CPF group. (**D**) Violin plot of the expression of circadian rhythm genes in the main cell types of the CPF group.

We selected a total of eight circadian clock genes (*Bmal1*, *Clock*, *Cry1*, *Cry2*, *Per1*, *Per2*, *Per3*, and *Rora*). The tSNE map was used to cluster and distinguish the expression patterns of circadian rhythm genes in the hypothalamus of the CPF and CD groups, as shown in Figure 5A,B. Compared to the CD group, we found that circadian clock genes, including *Clock*, *Cry2*, *Per1*, *Per2*, *Per3*, and *Rora*, had a relatively higher expression ratio in the CPF group. Among them, the percent expressed ratio and average expression of the rhythm gene *Per1* in cluster7 of the CD group were the highest (Figure 5C). In addition, the rhythm gene *Per3* also displayed a relatively higher expression ratio in cluster0, which primarily consisted of astrocytes.

The violin plot clearly shows the expression of circadian rhythm genes in each cell cluster in the hypothalamus of mice after CPF supplementation (Figure 5D). According to our experimental results, the expression effects on the rhythm genes *Per3* and *Rora* were more remarkable than on the others. It should be noted that the expression levels of *Clock*, *Cry1*, *Cry2*, *Per1*, *Per2*, *Per3*, and *Rora* in cluster0, cluster3, cluster6, cluster7, and cluster8 were more significant than in other cell clusters. In addition, results showed that astrocytes were highly enriched in cluster0 and cluster3, oligodendrocytes were enriched in cluster6 and cluster8, and microglia in cluster7. In addition, the rhythm gene *Bmal1* had a notable expression level only in the astrocytes.

### 3.8. CPF Supplementation Regulated the Different Expression Genes (DEGs) Involved in Myelination and Neurodegenerative Diseases

**Figure 6:**
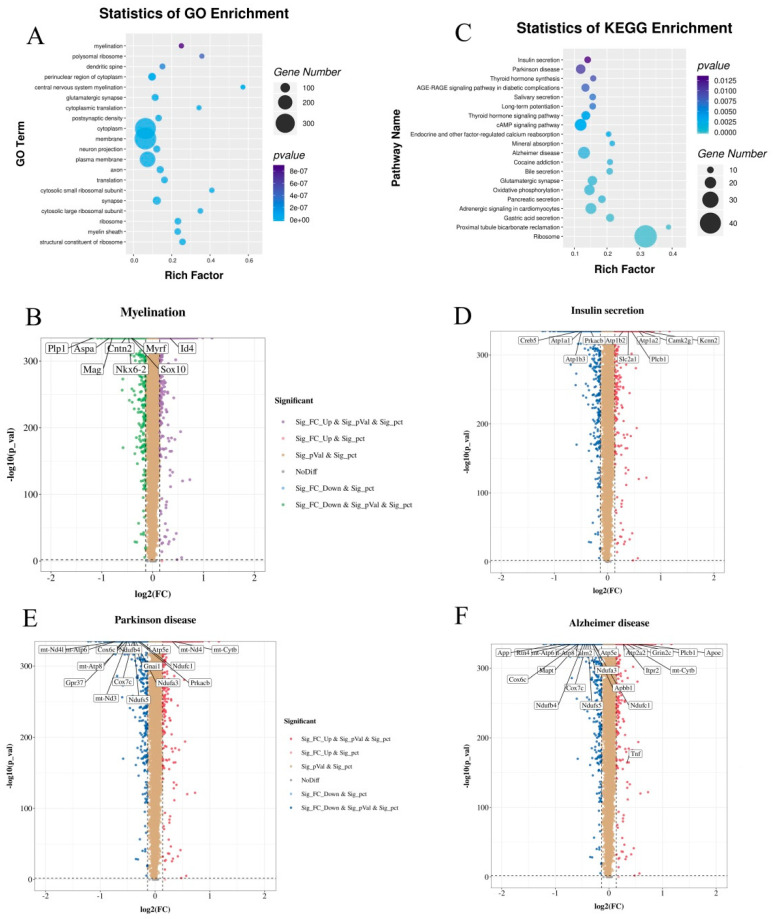
Effects of CPF intervention on gene enrichment pathways in the hypothalamus: (**A**) GO classification analysis of DEGs in the hypothalamus between the CD and CPF groups. (**B**) The regulation of CPF intervention on DEGs involved in myelination of the CNS. (**C**) KEGG classification analysis of DEGs in the hypothalamus between the CD and CPF groups. (**D**) The regulation of CPF intervention on DEGs involved in insulin secretion. (**E**) The regulation of CPF intervention on DEGs involved in Parkinson disease. (**F**) The regulation of CPF intervention on DEGs involved in Alzheimer disease.

To better understand how CPFs affect the biological functions of brain cells, we performed a transcriptome analysis of the different expression genes (DEGs) after 4 weeks of CPF intervention under CD treatment. As a result, we found that the CPF supplementation led to 361 up-regulated DEGs and 384 down-regulated DEGs. A Gene Ontology (GO) database analysis revealed that these genes might be involved in myelination, polysomal ribosomes, dendritic spines, central nervous system myelination, membranes, etc. (Figure 6A). It is worth noting that CPF intervention may subserve myelination via regulating DEGs, especially the myelin in the CNS. In addition, the volcano distribution diagrams show the regulation of CPF intervention on DEGs involved in myelination of the CNS, including one up-regulated and seven down-regulated (Figure 6B). Efficacy data support the conclusion that myelination increases the speed of conduction and synchrony of neural signals and plays a crucial role in major mental disorders, which may underlie mood symptoms and deficits in cognitive and psychosocial functioning [37,38].

Furthermore, Figure 6C displays the enrichment pathways of DEGs mapped to the KEGG database. The results demonstrate that “insulin secretion” and “Parkinson disease” were the most important DEG pathways. In addition, these DGEs were also involved in the “Alzheimer disease” pathway. The expression of genes involved in these three pathways is shown in Figure 6D–F. Among them, the “insulin secretion” pathway contains 6 up-regulated genes and 4 down-regulated genes, the “Parkinson disease” pathway includes 2 up-regulated genes and 14 down-regulated genes, and the “Alzheimer disease” pathway embodies 7 up-regulated genes and 14 down-regulated genes. Taken together, these results highlight that CPF supplementation has a supportable role in ameliorating neurodegenerative diseases in vivo.

## 4. Discussion

Circadian rhythms allow the host to synchronize its behavior and physiology with regular and predictable routines, including sleep–wake cycles and times of food consumption [39]. However, in modern times, the disruption of the circadian rhythm threatens the health of the host and increases the risk of a series of diseases. Previous studies suggested that the gut microbiota was tightly interrelated with its host circadian rhythm and exhibited circadian rhythmic oscillation. In some instances, the disruption of circadian feeding behavior contributed to a multi-faceted disruption of microbiota diurnal rhythmicity (for example, *Mucispirillum schaedleri*), thereby generating a temporal de-synchronization of circadian liver functions [40]. Enterobacter aerogenes has also been reported to have expressed circadian patterns of swarming and motility under an in vitro neurohormone melatonin intervention [41]. The results of the present study demonstrate that deliberate intervention to disrupt the circadian rhythm of mice can contribute to the perturbation of the gut microbiota in mice, including by up-regulating the ratio of F/B; increasing the relative abundance of *Parabacteroides*, *Clostridiales*, *Ruminiclostridium*, and *Lachnospiraceae*; and down-regulating the relative abundance of *Muribaculaceae* (the phenotypic biomarkers of the CT group).

In the present study, we demonstrated a range of beneficial effects of CPF supplementation on the gut microbiota homeostasis by using a mouse model with induced circadian rhythm disruption. By using a 16S rDNA gene sequencing analysis, after CPF supplementation, we found that the abundance of phylum *Firmicutes* significantly decreased and the abundance of phylum *Bacteroidetes* and *Verrucomicrobia* significantly increased. This finding is consistent with the report that ingesting flavonoids extracted from *Fructus Aurantii* reduces levels of intestinal *Firmicutes* and increases *Bacteroidetes* levels [42]. The ratio between *Firmicutes* and *Bacteroidetes*, a typical marker of gut dysbiosis and implicated in a predisposition to disease states, was significantly ameliorated by CPF supplementation in mice with circadian rhythm disruption. Importantly, *Bacteroidetes* is the predominant phylum in the human gut microbiota [43]. Microbiota belonging to the phylum *Bacteroidetes* were significantly associated with hippocampal proinflammation, and cognitive behavior index, which might contribute to the prevention of cognition decline [44].

Further analysis showed that the abundance of *Akkermansia muciniphila* in the phylum *Verrucomicrobia* increased, while the abundance of *Clostridiales* and *Ruminiclostridium* in the phylum *Firmicutes* displayed a significant upward trend in CPF-fed mice. Notably, our research demonstrated that CPF supplementation prevented a major gut microbiota shift and reversed the increase in major gut microbiota caused by circadian rhythm disruption. Specific gut bacterial species contribute to each of the behavioral domains, such as *Akkermansia muciniphila*. Nowadays, it is accepted that *Akkermansia muciniphila* is a kind of probiotic commonly appearing in the human gut [42]. *Akkermansia muciniphila* is a mucin-degrading bacterium that depends largely on mucins as an energy source and, in turn, stimulates goblet cells to produce mucus. The enhanced thickness of the mucus layer is conducive to improving the integrity of the intestinal barrier and protecting against the invasion of the epithelium by harmful bacterium [45]. A study demonstrated that *Akkermansia muciniphila* increased the levels of endocannabinoids that control inflammation, the gut barrier, and gut peptide secretion, and thus ameliorated metabolic disorders in diet-induced obese mice [46]. However, another research study also claimed that *Akkermansia muciniphila* caused significant accumulation in the group associated with multiple sclerosis (MS) [47]. Although these conflicting results remain, *Akkermansia muciniphila* may be a potential target for the early diagnosis or treatment of related diseases.

Next, we intended to explore the effects of CPFs on gut metabolites in mice with circadian rhythm disturbance. Our results showed that CPF supplementation preserved the levels of metabolites derived from the host diet and/or gut microbiota interactions. For instance, circadian rhythm disturbance groups exhibited decreases in daidzein and equol. Studies have demonstrated that daidzein is converted into equol; this takes place in the intestine via the action of reductase enzymes belonging to members of the gut microbiota, such as *Lactobacillus intestinalis* [48,49]. Increasing evidence suggests that equol exhibits superior blood–brain barrier (BBB) permeability and anti-neuroinflammatory activity in murine microglia and may be a potential nutraceutical for neuroprotection [50,51]. Thus, we speculate that circadian rhythm disturbance down-regulates the ability of gut microbiota to transform daidzein into equol. Intriguingly, our findings shed light on the possibility that CPF supplementation could promote the release of daidzein and equol, which were positively associated with *Akkermansia* and negatively correlated with *Ruminiclostridium.* Findings from this study support the conclusion that CPFs facilitate the release of daidzein via the gut microbiota, but further studies in vitro are warranted to prove this conclusion.

On the other hand, we found that a series of metabolites from the metabolism of dietary intake, such as lysoPC (16:0/18:1/20:4), decreased in mice exposed to continuous darkness but markedly increased after CPF intervention. Previous studies have suggested that a reduction in lysoPC levels could be used as a potential biomarker for some diseases. In some instances, researchers found that lysoPC levels that were significantly reduced contributed to worse glycemic control in diabetic patients with cardiovascular disease [52]. Another research study demonstrated that lysoPC activated glucose uptake and effectively lowered blood glucose levels in diabetes mouse models [53]. Furthermore, it has been reported that lysoPC can increase levels of the brain-derived neurotrophic factor and improve cognition and, thus, may be used as a potential treatment for neurological diseases [54]. More specifically, we also found that the feces of mice fed with CPFs displayed regulated levels of metabolites that are involved in neurological diseases, including up-regulated levels of acetylcarnitine, adenine, daidzein, luteolin, and xanthurenic acid and down-regulated levels of agmatine and ganoderic acid A. For example, Kazak et al. reported that acetylcarnitine increased the concentration of BDNF in the brain and effectively attenuated LPS-induced neuroinflammation in the brain [55]. Continuing with a holistic view, our results show that these differential metabolites were majorly concentrated in the “purine metabolism”, “tyrosine metabolism”, and “glycerophospholipid metabolism” pathways. However, whether CPFs directly participate in the formation of these metabolites or act via regulating the gut microbiota remains to be verified.

The adult vertebrate CNS mainly consists of neurons, astrocytes, oligodendrocytes, and microglia. In the present study, we found that astrocytes were more highly enriched in the CPF group than in the CD group, followed by oligodendrocytes and microglia. The number of neurons was decreased. The proper development and function of the mammalian CNS depend critically on the activity of these glial cells. As active components of the tripartite synapse, astrocytes are involved in neuronal support by releasing chemical transmitters via a process termed gliotransmission and play an essential role for the maintenance of the BBB and in neurovascular coupling [56]. Other evidence suggests that astrocytes acting through A1 receptors contribute to the regulation of sleep homeostasis, as well as cognitive deficit effects that arise from circadian rhythm disruption [57]. Oligodendrocytes are the neuroglial cells responsible for myelin sheath formation in the CNS [58]. It is worth noting that the DEGs affected by CPF intervention were mainly related to myelination, including one that was up-regulated and seven that were down-regulated. We speculate that a possible explanation for this is that CPF intervention may mediate the key genes of myelination in oligodendrocytes and then subserve myelination. Myelination is a remarkable example of cell differentiation that ensures fast signal propagation and the synchrony of neural signals [59]. Abnormalities in myelination can impair signal propagation along the axon and lead to nerve damage, thus, they cause deviations from optimal brain function in major mental disorders [60]. Microglia, serving as resident innate immune sentinels of the CNS, are capable of orchestrating potent inflammatory responses and contributing to brain development under normal conditions [35].

Furthermore, we analyzed the expression of circadian clock genes (*Bmal1*, *Clock*, *Cry1*, *Cry2*, *Per1*, *Per2*, *Per3*, and *Rora*) involved in the circadian rhythm in the CPF and CD groups. Our findings revealed that CPF supplementation significantly up-regulated the expression of circadian rhythm genes in different cells, which implies that CPFs could ameliorate circadian rhythm disruption. With respect to the overall gene expression, KEGG pathway analysis showed that the DEGs between the CPF and CD groups were involved in the “Alzheimer disease” and “Parkinson disease” pathways. CPF supplementation markedly up-regulated the key genes involved in these two pathways.

## 5. Conclusions

In conclusion, the present data show that CPF supplementation altered the specific composition and function of gut microbiota and metabolites induced by circadian rhythm disruption. CPFs affected the intestinal microbiota such that it repaired the intestinal environment and modulated the release of beneficial metabolites derived from the host’s diet and gut microbiota interactions, especially metabolites related to myelination in brain cells, anti-neuroinflammatory activity, and neuroprotective effects. In addition, single-cell RNA-seq revealed that CPF supplementation significantly up-regulated the expression of circadian rhythm genes and modulated the expression of genes associated with myelination and neurodegenerative diseases. Altogether, these findings highlight that CPFs may represent a promising dietary therapeutic strategy for treating the dysfunction induced by circadian rhythm disruption.

## Figures and Tables

**Table 1:** The α-diversity of gut microbiota among the three groups.

Groups	OTUs	Chao 1	Shannon	Simpson
CT	368.67 ± 20.87 ^c^	369.38 ± 31.24 ^c^	5.50 ± 0.14 ^b^	0.90 ± 0.02 ^a^
CD	236.33 ± 18.55 ^a^	236.33 ± 18.55 ^a^	4.87 ± 0.07 ^a^	0.96 ± 0.01 ^b^
CPF	278.02 ± 15.72 ^b^	278.56 ± 15.64 ^b^	5.48 ± 0.11 ^b^	0.91 ± 0.02 ^a^

Different letters indicate significant differences (*p* < 0.05) among different time points.

## Data Availability

Not applicable.

## Supplementary Materials

The following supporting information can be downloaded at: https://www.mdpi.com/article/10.3390/nu14112308/s1, Figure S1: The effect of CPF intervention on gut microbiota composition at the phylum level: (A) Difference analysis of gut microbiota composition at the phylum level between the CD and CT groups. (B) Difference analysis of gut microbiota composition at the phylum level between the CD and CPF groups. (C) The alternation of the microbial taxa of the CPF group at four weeks; Figure S2: (A) The unsupervised PCA analysis. (B) PCA model scores plot between the CD and CPF groups. (C) The supervised PLS-DA analysis between the CD and CPF groups. (D) Volcano plots of the different metabolites in the CD and CT groups. (E) Volcano plots of the different metabolites in the CD and CPF groups; Figure S3: The subgroups’ structural differences in cells for the three groups: (A) CT group. (B) CD group. (C) CPF group.
