# Supplementary figures and images for: The Modulatory Effect of Cyclocarya paliurus Flavonoids on Intestinal Microbiota and Hypothalamus Clock Genes in a Circadian Rhythm Disorder Mouse Model
Source: Nutrients. 2022 May 31;14(11):2308. doi: 10.3390/nu14112308 (PMC9182649; doi:10.3390/nu14112308)

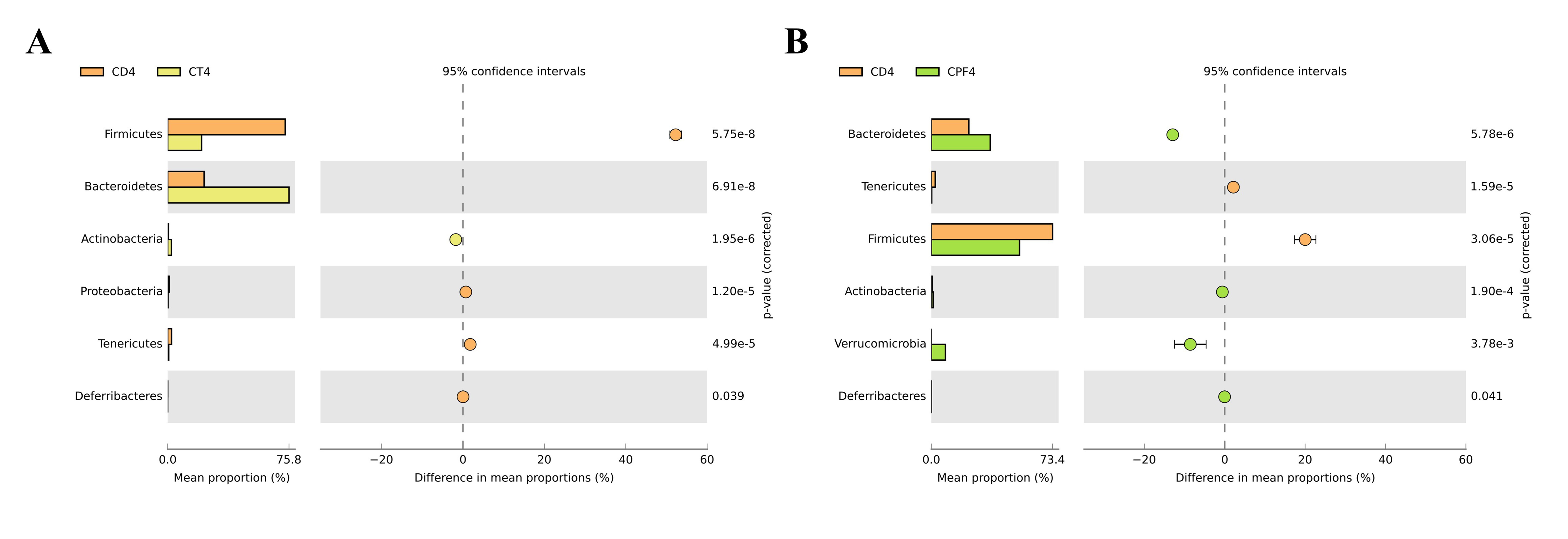

Supplement: Supplementary file 1 [file nutrients-14-02308-s001.zip › Figure S1.jpg]

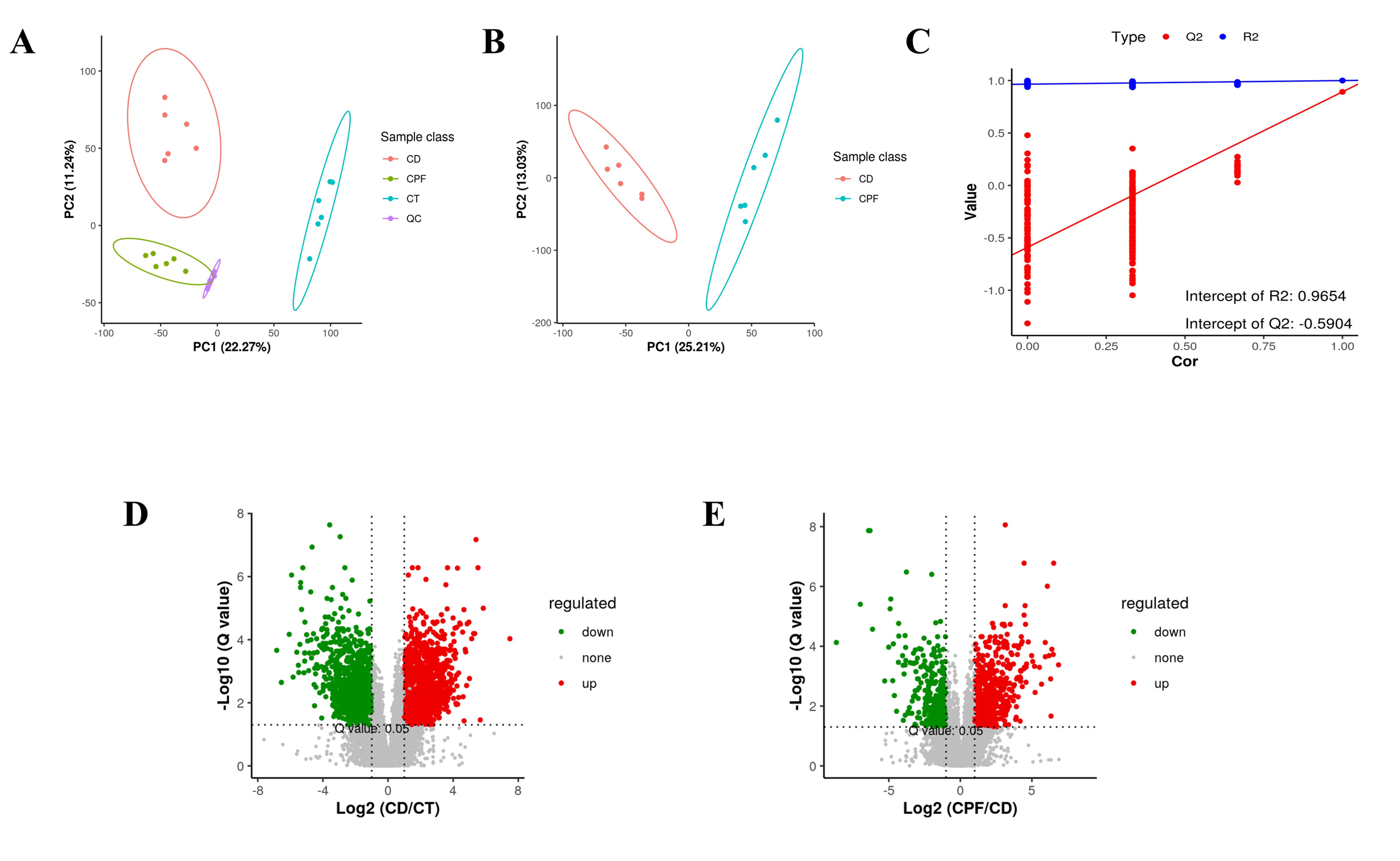

Supplement: Supplementary file 1 [file nutrients-14-02308-s001.zip › Figure S2.jpg]

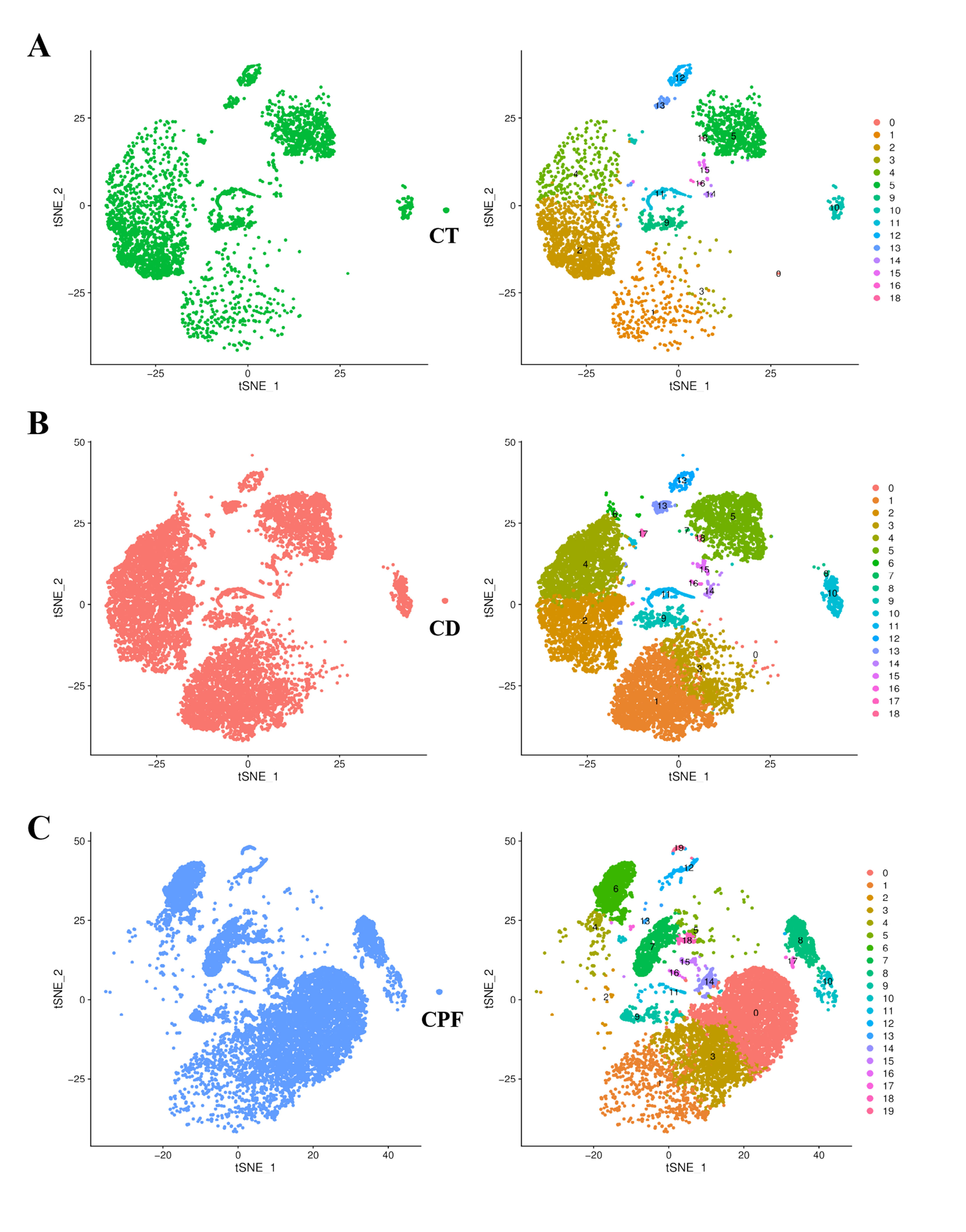

Supplement: Supplementary file 1 [file nutrients-14-02308-s001.zip › Figure S3.jpg]
